# Oncolytic virus-based suicide gene therapy for cancer treatment: a perspective of the clinical trials conducted at Henry Ford Health

**DOI:** 10.1186/s41231-023-00144-w

**Published:** 2023-04-10

**Authors:** Shivani Thoidingjam, Sushmitha Sriramulu, Svend Freytag, Stephen L. Brown, Jae Ho Kim, Indrin J. Chetty, Farzan Siddiqui, Benjamin Movsas, Shyam Nyati

**Affiliations:** 1 Department of Radiation Oncology, Henry Ford Health, 1 Ford Place, 5D-42, Detroit, MI 48202 USA; 2 College of Human Medicine, Michigan State University, East Lansing, MI 48824 USA

**Keywords:** Cancer gene therapy, Oncolytic adenovirus, Suicide gene therapy, Adenoviral gene therapy

## Abstract

Gene therapy manipulates or modifies a gene that provides a new cellular function to treat or correct a pathological condition, such as cancer. The approach of using gene manipulation to modify patient’s cells to improve cancer therapy and potentially find a cure is gaining popularity. Currently, there are 12 gene therapy products approved by US-FDA, EMA and CFDA for cancer management, these include Rexin-G, Gendicine, Oncorine, Provange among other. The Radiation Biology Research group at Henry Ford Health has been actively developing gene therapy approaches for improving clinical outcome in cancer patients. The team was the first to test a replication-competent oncolytic virus armed with a therapeutic gene in humans, to combine this approach with radiation in humans, and to image replication-competent adenoviral gene expression/activity in humans*. *The adenoviral gene therapy products developed at Henry Ford Health have been evaluated in more than 6 preclinical studies and evaluated in 9 investigator initiated clinical trials treating more than100 patients. Two phase I clinical trials are currently following patients long term and a phase I trial for recurrent glioma was initiated in November 2022. This systematic review provides an overview of gene therapy approaches and products employed for treating cancer patients including the products developed at Henry Ford Health.

## Gene therapy for cancer

### Introduction

U.S. Food and Drug Administration defines gene therapy “as a technique to modify the host genes, or manipulate the expression of a gene, or change the host cell biological properties for therapeutic use” There are several approaches to gene therapy. The most common approach is the replacement of a non-functional gene by insertion of a normal gene within the genome at a nonspecific site [1]. Mutated, defective genes that cause disease can be silenced or healthy genes that help prevent disease could be turned on via gene editing [2]. Other approaches include the swapping of the abnormal gene with a normal gene via homologous recombination, repair of the abnormal gene through selective reverse mutation, or altering the gene to regulate their expression [3]. Recent advancement in gene therapy also involves the introduction of genes in the patient’s cells are not missing but genes that offer therapeutic benefits [2]. Recently, gene therapy has made significant strides in cancer treatment, largely due to the advancement in the gene delivery techniques and vehicles (vectors), cargo that can be delivered, interaction of the host system to the vectors and the combined effect of the cargo with other therapeutic approaches [2, 4, 5].

Throughout history, infectious diseases such as severe acute respiratory syndrome coronavirus (SARS-CoV), plague, cholera and flu have had a major negative impact on human health. In modern times, infectious disease vectors have been reengineered to exploit their ability to infect human cells to correct or treat human disease. Cancer gene therapy has developed from strategies against monogenic conditions and diseases such as sickle-cell anemia, neuro- muscular disease and inherited blindness [6, 7]. A non-exhaustive list of clinical trials that utilize divergent gene therapy approaches for cancer treatment are presented in Table 1. Commonly used anti-cancer gene therapy strategies are immunotherapy, gene silencing, oncolytic virotherapy, gene directed-enzyme prodrug therapy (i.e. suicide gene therapy), among others [8]. Success of clinical studies led to approval of numerous gene therapy products for cancer treatment (Table 2). Herein we review some of the gene therapy approaches and gene therapy products, with special focus on oncolytic virus-based suicide gene therapy for cancer treatment.

**Table 1:** Cancer clinical trials utilizing different gene therapy approaches^a^

ClinicalTrials.gov Identifier	Condition/disease	Gene delivery method	Treatment	Status	First posted	Sponsor
NCT00116597	Pancreatic cancer	Adenovirus (Replication competent)	Theragene (Ad5-yCD/mutTKSR39rep-ADP) in combination with standard chemotherapy and radiation therapy	Phase 2: Recruiting (*n* = 12)	February 4, 2021	Seoul National University Bundang Hospital
NCT04486833	Lung Cancer	Lipid nanoparticles	Reqorsa (quaratusugene ozeplasmid, or GPX-001 is a lipid nanoparticle encapsulating a DNA plasmid with the TUSC2 tumor suppressor gene) in combination with Osimertinib	Phase 1/2: Recruiting (*n* = 92)	July 27, 2020	Genprex, Inc
NCT03603405	Glioblastoma Multiforme (GBM) and Anaplastic Astrocytoma (AA)	Adenovirus	ADV/HSV-tk (gene therapy)	Phase 1/2: Recruiting (*n* = 62)	July 27, 2018	The Methodist Hospital Research Institute
NCT01952730	Colorectal Cancer	Adenovirus	GVAX: Autologous, lethally irradiated colorectal cancer cells modified to produce GM-CSF	Phase 1: Recruiting (*n* = 15)	September 30, 2013	Massachusetts General Hospital
NCT02797470	HIV-related lymphoma	Lentivirus	Autologous Hematopoietic Stem Cell (Lentivirus Vector CCR5 shRNA/TRIM5alpha/TAR Decoy-transduced) Transplantation	Phase 1/2: Recruiting (*n* = 18)	June 13, 2016	AIDS Malignancy Consortium
NCT02280811	HPV-Associated Cancers	Retrovirus	Patient derived WBC genetically engineered to express TCR that will target the HPV-16 E6 oncoproteins constitutively expressed on HPV 16 + cancer cells	Phase 1/2: Completed (*n* = 12)	November 2, 2014	National Cancer Institute (NCI)
NCT03544723	Solid Tumors	Adenovirus	Adenoviral p53 + immune checkpoint inhibitors in patients with recurrent or metastatic cancers	Phase 2: Recruiting (*n* = 40)	June 4, 2018	MultiVir, Inc
NCT02858310	HPV-Associated Cancers	Retrovirus	Patient derived WBC genetically engineered to express TCR that will target the E7 proteins expressed by HPV + cancer cells	Phase 1/2: Recruiting (*n* = 180)	August 8, 2016	National Cancer Institute (NCI)
NCT02337985	AIDS-Related Non-Hodgkin Lymphoma	Lentivirus	Chemotherapy followed by transplantation of Hematopoietic Stem/Progenitor Cells transduced with Lentivirus encoding Multiple Anti-HIV RNAs	Phase 1: Recruiting (*n* = 10)	January 14, 2015	City of Hope Medical Center
NCT03541928	High-risk Prostate Cancer Prostate Cancer	Herpes Simplex Virus (Replication defective)	HSV-tk + valacyclovir gene therapy in combination with androgen deprivation therapy, brachytherapy, external beam radiotherapy, and prostatectomy	Phase 2: Recruiting (*n* = 60)	May 31, 2018	The Methodist Hospital Research Institute
NCT04911166	Non-small Cell Lung Cancer	Adenovirus (Replication defective)	Combination atezolizumab in combination with Interleukin-12	Phase 1: Recruiting (*n* = 16)	June 2, 2021	The Methodist Hospital Research Institute
NCT00004038	Breast Cancer	Adenovirus (Replication defective)	Adenovirus p53 (Ad5CMV-p53) gene in combination with chemotherapy	Phase 1: Completed (*n* = 20)	May 20, 2004	National Cancer Institute (NCI)
NCT03281382	Metastatic Pancreatic Cancer	Adenovirus (Replication competent)	Adenovirus-mediated cytotoxic and IL-12 gene therapy (Ad5-yCD/mutTKSR39rep-hIL12) in combination with chemotherapy	Phase 1: Completed (*n* = 12)	September 13, 2017	Henry Ford Health System
NCT00583492	Prostate Cancer	Adenovirus (Replication competent)	Replication-competent adenovirus-mediated suicide gene therapy (Ad5-yCD/mutTKSR39rep-ADP) in combination with intensity modulated radiotherapy (IMRT)	Phase 2: Completed (*n* = 44)	December 31, 2007	Henry Ford Health System
NCT02555397	Prostate Cancer	Adenovirus (Replication competent)	Oncolytic adenovirus-mediated cytotoxic and IL-12 gene therapy (Ad5-yCD/mutTKSR39rep-hIL12)	Phase 1: Active, not recruiting (*n* = 15)	September 21, 2015	Henry Ford Health System
NCT03281382	Metastatic Pancreatic Cancer	Adenovirus (Replication competent)	Oncolytic adenovirus mediated cytotoxic and IL-12 gene therapy (Ad5-yCD/mutTKSR39rep-hIL12) in combination with chemotherapy	Phase 1: Completed (*n* = 12)	September 13, 2017	Henry Ford Health System
NCT00005025	Fallopian Tube Cancer Ovarian Cancer Primary Peritoneal Cavity Cancer	Herpes Simplex Virus (Replication defective)	Herpes simplex thymidine kinase (HSVtk) vector producer cells (VPC) followed by ganciclovir	Phase 2: Unknown (*n* = 14–20)	May 5, 2003	John Stoddard Cancer Center at Iowa Methodist Medical Center
NCT00003450	Ovarian Cancer Peritoneal Cavity Cancer	Adenovirus (Replication defective)	Intraperitoneal adenoviral p53 (Ad5CMV-p53) gene therapy	Phase 1: Completed (*n* = 15–20)	December 5, 2003	University of Texas Southwestern Medical Center
NCT02894944	Pancreatic Cancer	Adenovirus (Replication competent)	Replication-competent Adenovirus-mediated Double Suicide Gene Therapy (Theragene®,Ad5-yCD/mutTKSR39rep-ADP) in combination with chemotherapy	Phase 1: Completed (*n* = 9)	September 9, 2016	Seoul National University Hospital
NCT01517464	Neoplasm	Liposome	Liposomes encapsulating RB94 gene (plasmid DNA) and attached with tumor targeting anti-transferrin receptor single chain antibody fragment (TfRscFv)	Phase 1: Completed (*n* = 13)	January 25, 2012	SynerGene Therapeutics, Inc
NCT02340117	Metastatic pancreatic cancer	Liposome	Cationic Liposomes encapsulating human WT p53 (SGT-53) used in combination with chemotherapy	Phase 2: Recruiting (*n* = 28)	January 16, 2015	SynerGene Therapeutics, Inc
NCT05062980	Non-Small Cell Lung Cancer	Lipid nanoparticles	Reqorsa (quaratusugene ozeplasmid, or GPX-001 is a lipid nanoparticle encapsulating a DNA plasmid with the TUSC2 tumor suppressor gene) in combination with pembrolizumab (PD-1 blocking antibody) or docetaxel ± ramucirumab	Phase 1/2: Recruiting (*n* = 156)	September 30, 2021	Genprex, Inc
NCT00001328	Brain Neoplasm Neoplasm Metastasis	Retrovirus	NIH 3T3 cell line producing retroviral vector carrying HSV-tk gene injected into the tumors and used in combination with Cytovene (Ganciclovir Sodium)	Phase 1: Completed (*n* = 15)	November 4, 1999	National Institute of Neurological Disorders and Stroke (NINDS)
NCT00505271	Breast Cancer	Retrovirus (Replication incompetent)	Rexin-G (retroviral vector carrying a mutant form of the cyclin G1 gene)	Phase 1/2: Completed (*n* = 20)	July 23, 2007	Epeius Biotechnologies
NCT00005796	CNS tumors	Retrovirus	Patient derived CD34 + cells transduced with retroviral vector expressing human O6-methylguanine DNA methyltransferase and reinfused into the patients along with chemotherapy	Phase 1: Completed (*n* = 10)	April 28, 2004	Indiana University
NCT02806687	Pancreatic Adenocarcinoma	Endoscopic ultrasound guided intratumoral injection	Gene Therapy product CYL-02 with Chemotherapy (Gemcitabine)	Phase 2: Active, not recruiting (*n* = 68)	June 21, 2016	University Hospital, Toulouse
NCT04995536	B-Cell Non-Hodgkin Lymphoma	Intratumoral needle injection	CpG-STAT3 siRNA CAS3/SS3 (CAS3/SS3) in combination with localized radiation therapy	Phase 1: Recruiting (*n* = 18)	August 9, 2021	

^a^The non-exhaustive list includes commonly used gene therapy vectors

**Table 2:** Currently approved Gene Therapy Products for Cancer treatment

Tradename	Proper Name	Approved on	Approval agency	Marketing-authorisation holder	Indication/ Therapeutic area
REXIN-G	Retroviral Expression Vectors Bearing Inhibitory Genes	September 2003	U.S. Food & Drug Administration (US-FDA)	Epeius Biotechnologies	Metastatic cancers
GENDICINE	Recombinant human p53 oncolytic adenovirus	October 2003	China State Food & Drug Administration (CFDA)	Shenzhen SiBiono GeneTech	Head and neck cancer
ONCORINE	Recombinant Human Adenovirus Type 5 Injection	November 2005	China State Food & Drug Administration (CFDA)	Shanghai Sunway Biotech	Head and neck and esophagus cancer, Nasopharyngeal cancer, etc
PROVENGE	Sipuleucel-T	April 2010	U.S. Food & Drug Administration (US-FDA)	Dendreon Corporation	Treatment of metastatic castrate resistant (hormone refractory) prostate cancer
IMLYGIC	Talimogene laherparepvec	December 2015 December 2021	European Medicines Agency (EMA) U.S. Food & Drug Administration (US-FDA)	Amgen Europe B.V	Melanoma
KYMRIAH	Tisagenlecleucel	August 2017 August 2018	U.S. Food & Drug Administration (US-FDA) European Medicines Agency (EMA)	Novartis Pharmaceuticals Corporation	Relapsed B-cell acute lymphoblastic leukemia
YESCARTA	Axicabtagene ciloleucel	October 2017 August 2018	U.S. Food & Drug Administration (US-FDA) European Medicines Agency (EMA)	Kite Pharma EU B.V., NL	Relapsed or refractory large B-cell lymphoma
TECARTUS	Brexucabtagene autoleucel	July 2020 December 2020	U.S. Food & Drug Administration (US-FDA) European Medicines Agency (EMA)	Kite Pharma EU B.V., NL	Relapsed or refractory mantle cell lymphoma (MCL) or B-cell precursor acute lymphoblastic leukemia (ALL)
BREYANZI	Lisocabtagene maraleucel	February 2021	U.S. Food & Drug Administration (US-FDA)	Juno Therapeutics, Inc	Relapsed or refractory large B-cell lymphoma
ABECMA	Idecabtagene vicleucel	March 2021	U.S. Food & Drug Administration (US-FDA) and European Medicines Agency (EMA)	Celgene Corporation	Relapsed or refractory multiple myeloma
CARVYKTI	Ciltacabtagene autoleucel	February 2022	U.S. Food & Drug Administration (US-FDA)	Janssen Biotech, Inc	Relapsed or refractory multiple myeloma
ADSTILADRIN	nadofaragene firadenovec-vncg	December 2022	U.S. Food & Drug Administration (US-FDA)	Ferring Pharmaceuticals A/S	High-risk Bacillus Calmette-Guérin (BCG)-unresponsive non-muscle invasive bladder cancer (NMIBC)

### Immunotherapy

One gene therapy strategy is to utilize genetically modified viral particles (vp) to activate the immune system to kill cancer cells. Cell-therapy, on the other hand utilizes modified cells (autologous or allogenic) to detect and kill cancer cells. Second and third generation vaccines for several cancers such as lung, prostate, pancreatic and malignant melanoma have shown encouraging results in recent clinical trials [9]. Chimeric Antigen Receptor (CAR) T-cell therapy, CART has been used for the management of leukemia (both myeloid and lymphoid) and shown promising results [10–13]. (CAR) T-cell therapy is a type of immunotherapy that utilizes a patient’s own T-cells (cell therapy) modified to express a chimeric T-cell receptor (TCR) that can recognize antigens on cancer cells and subsequently lyse the cells [14–16]. This strategy utilizes the antigen binding ability of monoclonal antibodies (mAb) and the lytic and self-renewal capacity of T-cells [17, 18]. The TCR consists of an extracellular non-self antigen recognition domain of a mAb and an intracellular TCR derived stimulatory domain [9]. Recognition of target antigens induce stimulation/activation of CAR T-cells which can kill cancer cells directly or by enhancing the secretion of immune modulators like cytokines, interleukins, and growth factors [15]. Some of the currently approved CAR T-cell therapies-based gene therapy products are Kymriah (Tisagenlecleucel) [NCT02445248, NCT02435849] [19, 20] and Yescarta (Axicabtagene Ciloleucel) [NCT02348216] [21, 22]. Kymriah (Table 2) was the first CAR T-cell therapy-based product to be approved for use in relapsed B-cell acute lymphoblastic leukemia [23]. T-cells from patients are modified to encode a CAR consisting of an extracellular domain specific for CD19, a transmembrane hinge, and an intracellular CD3 domain [5, 24]. Kymriah approach binds to the surface antigen CD19 found on diffuse large B-cell lymphoma (DLBCL) and other B-cell lymphomas to initiate an antitumor response [25]. Phase II clinical trials in patients with refractory DLBCL showed high response rates [26]. A similar approach is utilized by Yescarta which has been used to treat aggressive non-Hodgkin lymphoma management [27]. Patient T-cells are modified to recognize CD19 antigen on cancer cells by infecting with a gamma-retrovirus [28]. The CAR consists of an extracellular CD19 binding domain and a cytoplasmic domain with CD28 and CD3-zeta co-stimulatory domains [28, 29]. Despite challenges, the future use of CAR T-cell therapy for improved cancer outcomes is promising [30, 31].

### Gene silencing

Gene silencing refers to the inactivation of a gene to prevent expression of their products, when used clinically it is with therapeutic intent [32]. In cancer therapy, gene silencing is achieved by knockdown of target genes in tumor cells using RNA interference (RNAi) or clustered regularly interspaced short palindromic repeats (CRISPR)/CRISPR‐ associated protein (Cas) gene editing [33]. RNAi employs non-coding RNAs (ncRNAs), microRNA (miRNA), small Interfering RNA (siRNA) and short hairpin RNA (shRNA) to degrade and cleave the mRNA before translation [34]. Examples of siRNA delivery systems in clinical trials are CALAA-01 for use in malignant melanoma, and ALN-VSPOI for use in liver cancer and solid tumors patients [35]. The two-component CRISPR/Cas system has become an important genome editing tool for cancer cell and gene therapy and has been used in many clinical trials for cancer [36–45]. Some of the potential targets for gene silencing in cancer cells are mutated tumor suppressor genes, oncogenes, genes involved in cancer progression, and drug-resistance. The gene silencing approach is associated with fewer side effects compared to conventional chemotherapy since the target genes are selectively silenced [46]. Gene silencing studies using animals to target important cell cycle proteins by siRNA have demonstrated the proof-of-concept antitumor effect [47]. Phase I trials for pancreatic cancer and liver cancer therapy using liposomal siRNA formulations are currently underway [48].

### Oncolytic virotherapy

**Fig. 1:**
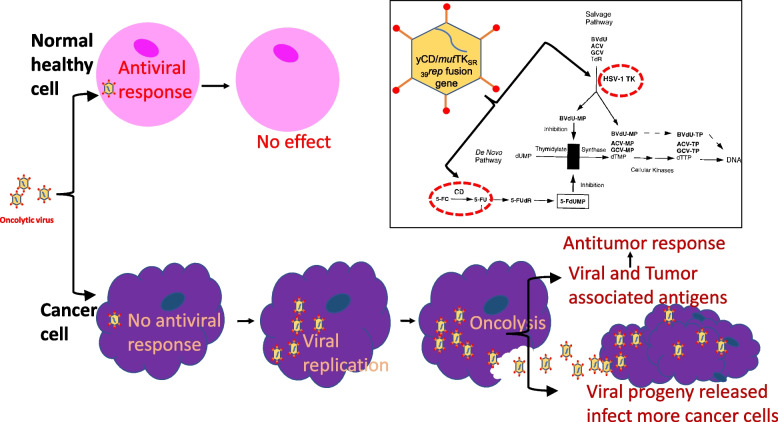
Mechanism of Oncolytic Virotherapy. When oncolytic viruses enter normal cells, the antiviral responses inhibit viral propagation. In cancer cells, aberrant signaling mechanisms lead to blockage of antiviral responses. Oncolytic viruses thus replicate and lyse host cancer cells that lead to release of virus in the immediate milieu. The released virus can infect nearby cancer cells which present with tumor-associated antigens and viral antigens that mediate anti-tumor responses. Only the replication-competent oncolytic viruses have the capability to replicate in cancer cells. Inset, the working model of the replication competent adenoviral gene therapy vector developed at Henry Ford Health that harbor two suicide genes. Gene products from these suicide genes (cytosine deaminase; CD, and thymidine kinase; TK) interfere with *de-novo* and salvage DNA replication pathways, that lead to enhanced cancer cell-killing when combined with cytotoxic chemotherapy or radiotherapy

Oncolytic virotherapy (OV) is an emerging treatment modality in cancer therapy [49–51]. The approach holds potential for metastatic cancers with promising results shown by several vectors in phase I trials [52]. Oncolytic or cancer-killing viruses are used in OV. These are defined as natural or genetically engineered viruses that can replicate selectivity in cancer cells (oncotropic) without damaging normal cells [49, 53, 54]. The mechanism of cancer cell killing by OV is illustrated in Fig. 1. Detailed mechanism is described by Li et. al., [55]. Viruses have unique properties that can be exploited for OV. Viruses have specificity for certain types of cells/cell receptors [56] such as an epithelial or neural cell, and they can also replicate in the host cell to self-amplify [57]. Thus, the need to infect every target cell at the time of treatment is eliminated. OV can exert anti-tumor effect in two ways; by infecting the cells directly, replicating inside and lysing them or by activating the immune system [58]. Oncolytic viruses carrying a gene of interest infect and introduce the exogenous genes into cells that otherwise lack them. Oncolytic viruses proliferate in the cancer cells to cause overwhelming viral infection and lysis which destroys the cancer cells. Lysis also leads to the release of the vp which infect nearby cells and metastases at distant sites in the body. The immune system is also activated due to the viral infection, leading to unmasking of tumor antigens, aiding the immune system in recognizing and attacking neoplasms [57]. Thus, oncolytic viruses can be used not only as a vector but also as an active cytotoxic and inflammatory agent [59].

OV for gene therapy employs two different classes of viruses (i) viruses that are naturally oncotropic, replicating preferentially in cancer cells or host cells with cancer-related mutations. They are often sensitive to the innate antiviral signaling or depend on host oncogenic signaling pathways in making them non-pathogenic in humans [60]. Most of the naturally occurring viruses have a tropism towards tumors/tumor cells because cancer cells are more adept than normal cells in avoiding immune surveillance and destruction, resisting apoptosis and translational suppression [54]. Naturally occurring oncotropic viruses thus replicate selectively in cancer cells without damaging normal cells. Examples include Parvoviruses, and Newcastle disease viruses. (ii) viruses that are genetically modified to confer oncolytic activity, improve the safety, tumor-specificity, potency, and decrease virus pathogenicity. The genes required for replication in normal cells but not in cancer cells are modified in these viruses so that they can replicate selectively in cancer cells. Some examples are adenoviruses, herpes simplex viruses (HSV), vesicular stomatitis viruses, vaccinia viruses and measles viruses [60] which are currently being tested in several clinical trials [61–64].

The adenoviruses are the most commonly used oncolytic viruses in gene therapy because of their tumor cell lytic and immune stimulation capacity [65]. Adenoviruses have non-enveloped icosahedral nucleocapsid and contain a double stranded DNA. There are 3 subtypes of gene products encoded by the ~ 36 kb length genome according to the transcription start time i.e., early, middle, and late stages. Among the various subtypes of adenoviruses, adenoviruses serotype 5 (Ad5) is the most used subtype. The recognition of specific surface receptors on tumor cells by the oncolytic adenovirus, such as the coxsackie and Ad receptor (CAR), causes their internalization into the tumor cells [66]. The viral genome translocate into the nucleus where the transcription of the early viral protein starts from the E1 region of the viral genome. The binding of the protein to Rb causes release of E2F transcription factor, resulting in the cell cycle activation and entry of the virus infected tumor cells into S phase. E2F release also activates viral genes, resulting in production of new virions that can infect more cells to lyse and produce more virions and continue the lytic cycle. The large loading capacity of the adenovirus gives an added advantage for loading therapeutic genes of interest [67]. While the adenovirus is infecting and replicating inside the tumor cells, the therapeutic genes are also continuously replicated (when under the control of a promotor) and expressed to bring about synergistic anti-tumor effect [67]. Oncolytic adenoviruses as a single therapy have not demonstrated a robust antitumor response in clinical trials. In contrast, the combination of oncolytic adenoviruses with other treatment modalities in cancer therapy have shown encouraging results [35]. ONYX-015 is an oncolytic adenovirus modified to replicate selectively in cancer cells deficient in p53, thus lysing and killing them. It was approved by the Chinese Food and Drug Administration in 2005 for use in refractory head and neck cancer along with cisplatin. Its use in the management of solid cancers has also being studied [68]. Ad5-D24, CG7870, recombinant H103, KH901, OBP-301, and Ad5-CD/TKrep are some of the other oncolytic adenoviruses assessed for use in cancer gene therapy [35]. ORCA-010, an oncolytic adenovirus serotype 5 (Ad5) is currently in clinical trial for use in prostate adenocarcinoma patients (phase I and II, first posted in September 2019, recruiting, *n* = 24) [69]. This trial, designed to evaluate the safety and tolerability of oncolytic adenovirus in humans for prostate cancer therapy, builds on the pioneering studies at Henry Ford Health in the late 1990s and early 2000s (described in detail below).

Some of the currently approved OV based gene therapy products are Gendicine, Oncorine, Imlygic and Rexin-G [5]. Among the viruses tested in preclinical trials, herpesvirus, adenovirus, and vaccinia virus have demonstrated favorable results [67]. In 2003, Gendicine (Ad5RSV-P53) was the first approved gene product for Head and Neck squamous cell carcinoma (HNSCC) [70]. This non-replicative adenoviral vector restores the expression of p53 in p53 mutated tumor cells. The Adenovirus E1 gene is replaced with the tumor suppressor p53 cDNA gene. Tumor cells administered with Gendicine show p53 expression that induce the antitumor response by activating the apoptotic pathway and inhibiting repair of damaged DNA and anti-apoptotic activity [71]. Records of 12 years of use of Gendicine in Head and Neck cancer in more than thirty thousand patients and more than thirty clinical trials have shown the exceptional safety profile, and significantly higher response rates when Gendicine is used in combination with chemotherapy or radiotherapy compared to conventional therapies alone [72]. In addition to Head and Neck cancer, Gendicine combined with chemotherapy or radiotherapy has also shown significantly longer progression-free survival time than conventional therapies alone in several other cancers [72].

Oncorine (rAd5-H101) was the first replicative, oncolytic recombinant adenovirus approved for use in treatment of nasopharyngeal cancer. It was designed to proliferate only in cancer cells and not normal cells. This was done by knocking out the adenovirus E1B 55 K gene. The gene product of E1B 55 K gene, E1B protein binds to and degrades p53 transcription factors, thereby preventing apoptosis in cells. The inactivation of the gene inhibits viral proliferation in normal cells with functioning p53 gene, while in cancer cells with mutant p53, these viruses replicate, package its genome, lyse the cell, and spread to new cells [73].

Imlygic (Talimogene Laherparepvec) was approved in 2015 in Europe for non-resectable metastatic melanoma. It is a genetically modified herpes simplex virus 1 (HSV-1) where both copies of the viral gene responsible for suppressing an immune response (ICP34.5) were removed and replaced with human GM-CSF. Imlygic lead to apoptosis of tumor cell with enhancement in antigen presentation and increased antitumor response [74].

Rexin-G (Mx-dnG1) is a tumor targeted replication-incompetent retroviral vector and the first injectable vector to be approved for metastatic cancers. The retrovirus bears a mutant cytocidal cyclin G1 gene, which is an important component of the cell cycle pathways. It binds to the abnormal signature proteins (SIG) in tumor cells via a SIG-binding peptide, thereby increasing its concentration in tumor cells. Once inside the cells, Rexin-G lead to apoptosis in cancer cells via the synthesis of cytocidal dnG1 proteins that inhibit the cell cycle in the G1 phase [75]. PVSRIPO is an oncolytic recombinant polio/rhinovirus which is currently in phase II clinical trials for safety and efficacy study in recurrent malignant glioma [76].

The advantages of OV over other therapy approaches include low probability of therapy resistance, ability to target multiple oncogenic pathways to kill cancer cells, selective replication in cancer cells, non-pathogenicity (minimal systemic toxicity), amplification of the vp with time in the host (replication competent OV) in contrast to chemotherapy which demonstrates reduction over time, and room for modifications for increased safety and efficacy [54]. One major disadvantage of OV is complexities associated with its efficient delivery. That includes pre-existence of anti-OV antibodies or induction upon multiple administrations, affinity to serum proteins and instability during circulation, minimal to no extravasation into the tumors, or liver sequestration [54, 60]. The limited efficacy of OV in solid tumors could be due to presence of physical barriers, tumor heterogeneity, and an immunosuppressive tumor-microenvironment (TME) [55]. Optimization of OV delivery approaches, converting the immunosuppressive TME to inflammatory TME, increasing efficacy and safety are some areas that still need significant improvement [53].

### Suicide gene therapy

In suicide gene therapy approach, a gene expressing a non-toxic enzyme is introduced into the cancer cells. This is followed by the systemic administration of a prodrug which is converted into a toxic compound by an enzyme produced by the newly introduced genes in the cancer cells. Thus, the cancer cells are selectively killed while the uninfected normal cells are relatively spared from damage. The genes used are of non-human origin, generally viral or bacterial genes. Of the several suicide gene systems identified, the cytosine deaminase/5-fluorocytosine and the herpes simplex virus/ganciclovir are widely used [29, 77]. This approach can also affect and kill neighboring tumor cells exhibiting a bystander effect due to toxic metabolite diffusion. The dying tumor cells can also release necrotic materials and tumor antigens into the circulation causing an immune system activation leading to killing of distant tumor metastases [35]*.*

## Gene therapy clinical trials at Henry Ford Health

**Fig. 2:**
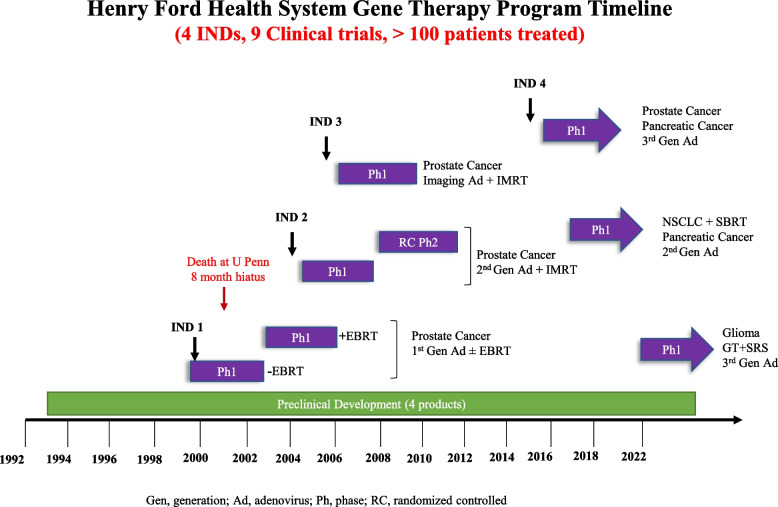
Timeline of Gene therapy vector development and initiation of investigator initiated clinical trials at HFH

Over the last two decades investigators at Henry Ford Health have developed four different gene therapy vectors. The replication competent adenoviral vectors developed have been designed to harbor two mutant suicide genes in addition to carrying a gene for enhanced cytolysis, imaging or for immunotherapy. The products have been evaluated in more than 6 preclinical studies [78–85]. The team has conducted 9 investigator-initiated phase I, phase I/II clinical trials [86–94] (Fig. 2) including a trial that remains open for long term follow up of recurrent prostate cancer patients treated with suicide gene therapy including IL-12 (Table 3). The team recently initiated an investigator-initiated phase I trial that aims to identify maximum tolerated dose (MTD) and safety of adenoviral gene therapy in combination with cytotoxic prodrugs and fractionated radiosurgery (fSRS) in adults with recurrent glioma (Table 3).

**Table 3:** Cancer Gene Therapy Clinical Trials conducted at HFH using HFH developed vectors

IND	IRB	Virus	Virus Dose	Rx	Site	Patient #	Phase	Status
11,253	4809	CD/TKrep-ADP	1 × 10^12^	Arm1: GT + IMRT 80 Gy, Arm2: IMRT 80 Gy	Prostate	44	II	Closed 11/23/21
11,253	2800	CD/TKrep-ADP	1 × 10^11^ 1 × 10^12^	GT + IMRT 74 Gy	Prostate	9	I	Closed, 10/14/14
16,536	9829	CD/TKrep-IL-12	1 × 10^10^ 3 × 10^10^ 1 × 10^11^ 3 × 10^11^ 1 × 10^12^	GT only	Prostate	15	I	Open*
16,536	11,260	CD/TK-rep-IL-12	1 × 10^11^ 3 × 10^11^ 1 × 10^12^	GT + Chemo	Pancreas	12	I	**Completed**
12,786	3828	CD/TKrep-hNIS	1 × 10^11^ 1 × 10^12^	GT + IMRT 76 Gy	Prostate	19	I	Closed, 3/11/14
8436	361	CD/TKrep	1 × 10^10^ 1 × 10^11^ 1 × 10^12^	GT only	Prostate	16	I	Closed, 6/25/12
9852	588	CD/TKrep	1 × 10^12^	GT + EBRT 70–74 Gy	Prostate	15	I	Closed, 3/11/14
11,253	15,295	CD/TKrep-ADP	1 × 10^11^ 3 × 10^11^ 1 × 10^12^	GT + SRS (8 Gy*3)	Glioma	9–18	I	**Open/recruiting**

Open*: Not recruiting new patients, long term follow-up only. IRB not yet officially closed

Virus-based suicide-gene therapies are based primarily on the premise that tumor cells expressing the suicide genes will be rendered sensitive to specific pharmacologic agents thereby providing a local cytotoxic effect and improving the therapeutic index [78, 84, 85, 95–103]. Delivery of the virus to the tumor is usually accomplished by direct intra-tumoral or systemic injection of a viral vector containing the suicide gene.

Two suicide genes that have been evaluated in preclinical models and in the clinic are the *E. Coli* cytosine deaminase (CD) and herpes simplex virus thymidine kinase (HSV-1 TK). Cytosine deaminase (CD) can deaminate the innocuous prodrug 5-fluorocytosine (5-FC), producing locally high concentrations of 5-fluorouracil (5-FU), a widely used chemotherapeutic agent. The cytotoxic effect of 5-FU follows its conversion to 5-fluoro-deoxyuridylic monophosphate (dUMP), which irreversibly inhibits thymidylate synthase, resulting in the inhibition of the de novo pathway of DNA synthesis. Thymidine kinase (TK) from herpes simplex virus type (HSV-1) phosphorylates a variety of nucleoside analogues, such as ganciclovir (GCV), to their nucleoside monophosphate derivatives. Once phosphorylated, these analogues are converted by endogenous cellular kinases into their corresponding nucleoside triphosphates, which inhibit the salvage pathway of DNA replication by chain termination. Both the CD/5-FC and HSV-1 TK/GCV suicide gene systems exhibit a bystander effect, which results in the destruction of neighboring tumor cells not expressing the suicide genes [100, 101, 104]. The three generations of lytic adenoviral vector developed at Henry Ford Health for suicide gene therapy and the preclinical and clinical trials using these products are described below.

### Ad5-CD/TK*rep* suicide gene therapy: first-generation adenoviral vector

The search for viral vectors that could enhance gene therapy and overcome the limitation of the replication defective ones led to the development of the first-generation adenoviral vector Ad5-CD/TK*rep* at Henry Ford Health. This vector was a replication competent, lytic adenovirus type 5 and could replicate in the tumor cells to increase the therapeutic efficacy per dose of virus administered. Compared to ONYX-01 (one of the first replication-competent, ElB-attenuated adenovirus 5) Ad5-CD/TK*rep* demonstrated improved efficacy and safety. This virus was also armed with a combined double enzyme-prodrug systems, a yeast cytosine deaminase (yCD) /5-FC and HSV1-tk/ganciclovir that caused enhanced cancer cell killing and sensitized cancer cells to radiation in vitro [78]. Kim *et. al*., first demonstrated the selective enhancement of radiation killing by the HSV1-tk/ganciclovir system *in-vitro* and *in-vivo*[105].

Subsequent in vivo studies using this virus proved that the virus by itself exhibited antitumor effects in mice with C33A tumors. Use of the virus with both prodrugs, but not single drugs led to improved therapeutic outcomes. The use of this virus with the two prodrugs and radiation led to tumor regression and complete tumor cure demonstrating the therapeutic potential of this triple combination approach in cancer gene therapy [79]. The safety and efficacy of using this virus with external beam radiation therapy (EBRT) was evaluated in a preclinical study using two mouse prostate cancer models in preparation for Phase I human clinical trials. Both prostate cancer models confirmed improved tumor control and reduced lymph node metastases using the triple combination of virus, prodrugs and EBRT compared to EBRT alone. The trimodal therapy was however associated with limited toxicity; toxicity was attributed to the combined Ad5-CD/TK*rep* adenovirus and EBRT, not the prodrugs. This study concluded that the double suicide gene therapy mediated by the Ad5-CD/TK*rep* adenovirus can be used to effectively enhance EBRT and was safe to use for human trials [80].

In a phase I study, which was the first ever human gene therapy trial using replication-competent virus to deliver genes for therapy, the efficacy of the Ad5-CD/TK*rep* adenovirus for treatment of locally recurrent prostate cancer was evaluated. The adenovirus was administered to 12 patients in 3 groups in escalating doses (10^10^, 10^11^, and 10^12^ vp) intra-prostatically followed by 5-fluorocytosine and ganciclovir prodrug therapy two days later for 1 week in groups 1–3. If no dose limiting toxicity (DLT) was observed, the study was to proceed to the next group. A fourth group with 4 patients was thus included in the study where they received 10^12^ vp followed by 5-fluorocytosine and ganciclovir prodrug therapy two days later for 2 weeks instead of one as in groups 1–3. No dose limiting toxicities were observed and the MTD of the adenoviral vector was not defined. Mild adverse effects were observed, with 44% patients demonstrating a 25% or more decrease in serum prostate-specific antigen (PSA) and 19% of patients exhibiting 50% or more decrease in serum PSA. The viral DNA was detected in the blood of patients up to 76 days post-viral injection. One year follow up indicated that two patients were negative for adenocarcinoma. This study concluded that the Ad5-CD/TK*rep* carrying the double suicide genes could be safely used in combination with 5-FC and GCV prodrug therapy in humans [86].

Another phase I trial studied the safety of using Ad5-CD/TK*rep*-prodrug therapy with conventional-dose three-dimensional conformal radiation therapy (3D-CRT) in patients with newly diagnosed intermediate- to high-risk prostate cancer. Virus was injected into the prostate gland under ultrasound guidance, followed by 5-fluorocytosine and valganciclovir prodrug therapy two days later for 1, 2 or three weeks depending on patient groups. The patients presented with mild or moderate adverse effects with no dose limiting toxicities. Acute urinary/gastrointestinal toxicities seen were associated with the conventional-dose 3D-CRT. The PSA half-life was found to be shorter in patients receiving prodrug therapy for more than a week compared to one week only and markedly shorter than patients receiving only conventional-dose 3D-CRT. This clinical trial demonstrated the safety of using Ad5-CD/TK*rep*-prodrug therapy with conventional-dose 3D-CRT [87].

### Ad5-yCD/*mut*TK_SR39_*rep*-ADP suicide gene therapy: second-generation adenoviral vector

Henry Ford Health investigators developed the Ad5-yCD/*mut*TK_SR39_*re*p-ADP oncolytic adenovirus, which was a second-generation replication competent adenovirus type 5. This second-generation adenovirus had an improved yeast cytosine deaminase (yCD) instead of bacterial cytosine deaminase and a mutant SR39 (Serine to Arginine) herpes simplex virus thymidine kinase (*mut*TK_SR39_), resulting in both the enzyme products having better catalytic activity [106, 107]. The virus also carried the 11.6-kDa Ad5 adenovirus death protein (ADP) gene which confers enhanced oncolytic activity. Thus, compared to the first-generation Ad5-CD/TK*rep* adenovirus, Ad5-yCD/*mut*TK_SR39_*rep*-ADP demonstrated enhanced chemo-radiosensitivity causing increased cell killing and greater tumor control in vitro and in preclinical studies human cancer models, respectively [85].

The efficacy and toxicity of combining Ad5-yCD/*mut*TK(_SR39_)*rep*-ADP adenovirus gene therapy and radiation was studied in a preclinical model of pancreatic cancer. Human pancreatic adenocarcinoma cells MiaPaCa-2 and PANC-1 were sensitive to the Ad5-yCD/*mut*TK_SR39_*rep*-ADP adenovirus harboring yeast cytosine deaminase (yCD) and herpes simplex virus thymidine kinase (HSV-1 TK(SR39) suicide gene systems. MiaPaCa-2 tumor bearing athymic mice survived longer when treated with Ad5-yCD/*mut*TK_SR39_*rep*-ADP-mediated adenovirus with radiation than mice treated with either modality alone. The HSV-1 TK gene in the Ad5-yCD/*mut*TK_SR39_*rep*-ADP adenovirus system can be imaged by positron emission tomography (PET) by administering positron-emitting substrates of HSV-1 TK such as 9-(4-[^18^F]-fluoro-3-hydroxymethylbutyl)guanine ([^18^F]-FHBG). Following intrapancreatic injection in dogs, [^18^F]-FHBG was detected in the pancreas but not in the other major organs evidencing little dissemination of the adenovirus to uninvolved tissues. This study confirmed that the Ad5-yCD/*mut*TK_SR39_*rep*-ADP adenovirus system can enhance the killing effect when used in combination with radiation and can be used for non-invasively imaging of the gene expression using PET substrates [81].

The safety of Ad5-yCD/*mut*TK_SR39_*rep*-ADP with radiation therapy was tested in phase I and II clinical trials in prostate cancer patients. Phase I toxicity and preliminary efficacy study of using Ad5-yCD/*mut*TK_SR39_*rep*-ADP with intensity-modulated radiotherapy (IMRT) resulted in low toxicity with 92% of adverse effects being considered mild or moderate, and one non-associated death [90]. The Phase II trial was a multicenter, prospective, randomized study using IMRT with or without oncolytic adenovirus-mediated cytotoxic gene therapy. Two-year prostate cancer biopsy results confirmed a 42% relative reduction in biopsy positivity in the arm that received Ad5-yCD/*mut*TK_SR39_*rep*-ADP with IMRT compared to the arm that received only IMRT. None of the patients exhibited hormone-refractory or metastatic disease, and there was no patient death [108]. In a longer follow up of five years, this protocol delayed the need of salvage therapy by two years and 88% of the patients were alive with no deaths reported due to prostate cancer [109]. The safety and success of these trials have encouraged the Henry Ford Health team to investigate this approach at other tumor sites.

### Ad5-yCD/*mut*TK_SR39_*rep*-mIL12 suicide gene therapy: third-generation adenoviral vector

Although the second-generation oncolytic adenoviral vector could control tumor locally, it was important to find new therapies to target metastatic tumors to further improve cancer patient survival. With the goal to improve adenoviral vector mediated gene therapy, the Henry Ford Health team developed the new strategy of adding a cytokine gene therapy (i.e., immunotherapy) to the Ad5-yCD/*mut*TK_SR39_*rep* adenoviral vector.

Cytokines play a major role in regulating both the innate and adaptive immunity [110]. Cytokines exert their anti-tumor effect by a direct anti-proliferative or pro-apoptotic activity, or by indirectly stimulating the cytotoxic activity of immune cells to target cancer cells [111]. Preclinical studies demonstrated favorable efficacies with granulocyte–macrophage colony stimulating factor (GM-CSF), interferon alpha (IFNα), interleukin (IL)-2, IL-12, IL-15, and IL-21 [112–119]. These preclinical studies, however, failed to translate into clinical trials due largely to their short half-lives and narrow therapeutic window. IFNα was the first cytokine approved for human cancer treatment (hairy cell leukemia; HCL) followed by approval of high-dose IL-2 (HDIL-2) for metastatic renal cell carcinoma (mRCC) and metastatic melanoma (MM). However, the toxicity and low response rate with HDIL-2 and IFN-α have limited their use in cancer therapy [111, 120, 121].

The oncolytic adenovirus (Ad5-yCD/*mut*TK_SR39_*rep*-mIL12) expressing the double suicide gene systems and mouse interleukin-12 (IL-12) was thus engineered at Henry Ford Health. This vector (Ad5-yCD/*mut*TK_SR39_*rep*-mIL12) was found to improve tumor control in both local and metastatic tumors in a preclinical TRAMP-C2 prostate adenocarcinoma mouse model to improve survival significantly compared to adenovirus lacking IL-12 (Ad5-yCD/*mut*TK_SR39_*rep*) [83]. Ad5-yCD/*mut*TK_SR39_*rep*-mIL12 led to increased production of IL-12 and IFN-γ in tumor and serum and better anti-tumor immune responses. For optimal activity of Ad5-yCD/*mut*TK_SR39_*rep*-mIL12, both adaptive and innate immunity were found to be required. This study demonstrated that the IL-12 addition could significantly improve the efficacy of locally (intratumorally) administered adenovirus mediated oncolytic gene therapy.

Additionally, a preclinical study of the combination of adenovirus-mediated cytotoxic pro-drugs and interleukin 12 (IL-12) gene therapy was carried out to support phase I clinical trials. The oncolytic adenovirus Ad5-yCD/*mut*TK_SR39_*rep*-mIL12 expressing two suicide genes and mouse IL-12 was intraprostatically administered in mice, followed by two weeks of 5-FC and GCV prodrug therapy [82]. This study showed mild side effects which resolved themselves or were unremarkable and demonstrated the tolerability of the Ad5-yCD/*mut*TK_SR39_*rep*-mIL12 oncolytic adenovirus in mice, supporting use in human trials [82].

A similar adenoviral vector was developed that harbored human IL-12 gene (Ad5-yCD/*mut*TK_SR39_*rep*-hIL12) and was subsequently used in an investigator-initiated phase I trial in men with locally recurrent prostate cancer after definitive radiotherapy [NCT02555397]. Ad5-yCD/*mut*TK_SR39_*rep*-hIL12 adenovirus was administered via intraprostatic injection at a single dose of 1 × 10^10^ to 1 × 10^12^ vp. The primary goal of the study was to determine the dose-dependent toxicity and MTD within 30 days from the first day of treatment. Secondary objective included PSA response and PSA doubling time, freedom from biochemical/clinical failure and quality of life over a period of two years, disease specific survival and overall survival (OS) over a period of 5 years. Preliminary results revealed that MTD was not reached even at the highest dose of 1 × 10^12^ vp [94]. Patients are being currently followed for long-term outcomes and results of this trial will be presented (manuscript in preparation).

In a similar approach, this 3^rd^-generation adenoviral vector was used in a phase I trial in metastatic pancreatic cancer patients [NCT03281382]. Increasing doses of Ad5-yCD/*mut*TK_SR39_*rep*-hIL-12 (1 × 10^11^, 3 × 10^11^, or 1 × 10^12^ vp) were administered into the primary pancreatic tumor using gastrointestinal ultrasonography into subjects. This was followed by 5-fluorocytosine (5-FC) therapy for 7 days after adenovirus injection. Chemotherapy (FOLFIRINOX or gemcitabine/albumin-bound paclitaxel) was initiated 21 days after adenoviral injection. The primary endpoint was toxicity through day 21. Most adverse events (AE; 94%) were grade 1/2 which did not require any medical intervention. Only two patients (out of twelve) had Ad5-yCD/*mut*TK_SR39_*rep*-hIL-12 DNA detected in their blood. Elevated levels of IL-12*,* IFN-γ, and CXCL10 was detected in patient serum in 42%, 75%, and 92% of the subjects, respectively. Immune cells were also found to be activated following administration of Ad5-yCD/*mut*TK_SR39_*rep*-hIL-12. The third cohort of patients, those receiving the highest dose of virus, showed a median survival of 18.1 (range, 3.5–20.0) months. The MTD of the treatment was not reached in the study [89].

One of the ways to improve adenoviral gene therapy is to develop methods to monitor the activity of these vectors following administration into patients. The Henry Ford Health team developed the Ad5-yCD/*mut*TK_SR39_r*ep*-hNIS adenoviral vector to track the spatial and temporal gene expression in vivo. This vector carries a human sodium iodide symporter (hNIS) reporter gene. The human sodium iodide symporter can catalyze uptake of negatively charged ions like iodide, technetium etc. and has several substrates that can be detected using CT, PET or SPECT imaging [122, 123]. Following intraprostatic injection of the Ad5-yCD/*mut*TK_SR39_*rep*-hNIS adenoviral vector in men, non-invasive imaging of NIS gene expression by Na^99m^TcO_4_ uptake in cells infected with the vector was performed by SPECT. Gene expression was detected in 7 of 9 patients with intensity peaking at 1–2 days post injection and detectability up to 7 days. Intraprostatic gene expression was seen but no evidence of the dissemination of the virus outside the prostate was found on whole body imaging. This study demonstrated that the viral vector activity can be monitored in humans non-invasively and safely [92].

Table 3 and Fig. 2 summarize and provide a timeline of the different gene therapy clinical trials conducted at Henry Ford Health. Henry Ford Health also participated/is participating in several sponsored gene therapy clinical trials for various cancers. These trials are summarized in Table 4.

**Table 4:** Gene Therapy Clinical Trials Conducted at HFH Using Externally Developed Products

ClinicalTrials.gov Identifier	Product used/Treatment	Cancer	Status^a^	Sponsor	Remarks
NCT04180215	HB-201 and HB-202: Replicating live-attenuated arenavirus vectors expressing HPV16 E6/E7 fusion protein	Head and Neck Squamous Cell Carcinoma and other solid tumors	Phase 1/ 2: Recruiting (*n* = 200)	Hookipa Biotech GmbH	HB-201 monotherapy and HB-201 and HB-202 alternating two-vector therapy were well tolerated. HB-201 and HB-202 alternating two-vector therapy showed superior immune and more robust antitumor response compared to monotherapy
NCT03491683	DNA medicines (INO-5401 and INO-9012) with programmed death-1 (PD-1) protein targeting antibody (Cemiplimab) and chemo-radiotherapy	Glioblastoma (GBM)	Phase 1/2: Active, not recruiting (*n* = 52)	Inovio Pharmaceuticals	The primary outcome of the trial will study percentage of participants with Adverse Events at 0–30 days, 6 months and up to 24 months after the last dose of study treatment
NCT00676507	Lucanix™ vaccine therapy	Advanced Non-small Cell Lung Cancer (NSCLC)	Phase 3: Completed (*n* = 532)	NovaRx Corporation	The vaccine was well tolerated with no difference in the survival and PFS of patients in the two groups. However, an improved survival in Lucanix™ treated group was suggested in patients randomized within 12 weeks after the front- line chemotherapy was complete and in patients who had received radiation prior to vaccine treatment
NCT00050388	Allovectin-7®	head and neck cancer	Phase 3: Completed	Vical	Despite exhibiting safety and well tolerability with partial or complete tumor response in 33% patients in phase 1 and 2 trials, phase 3 trials failed to achieve their primary and secondary outcomes of tumor response and overall survival compared to chemotherapy in metastatic malignant melanoma patients
NCT01156584	Toca 511 or vocimagene amiretrorepvec: a nonlytic replicating γ-retroviral vector that encode for an optimized yCD transgene	Grade III/IV Gliomas	Phase 1: Completed (*n* = 54)	Tocagen Inc	A subgroup with both isocitrate dehydrogenase 1 (IDH1) mutant and wild-type tumors was identified for the follow up phase 3 study. This subgroup showed 21.7% durable response rate and follow-up for responders for median duration was 35.7 + months. After 33.9 + to 52.2 + months of administration of Toca 511, all responders were alive and remained in response, indicating a positive correlation between durable response and overall survival
NCT01156584	Toca 511	Recurrent high-grade glioma (rHGG)	Phase 1: Completed (*n* = 54)	Tocagen Inc	Depending on the group, patients were given Toca 511 intratumorally via transcranial injection or intravenously daily for 3 and 5 days. 3 to 4 weeks after Toca 511 administration, Toca FC was given and repeated approximately after every 6 weeks until end of study. All the patients after the end of the study were eligible to enroll in a long-term continuation protocol, which enabled additional dosing with Toca FC and permitted the long-term safety and survival data to be collected
NCT02414165	Toca 511 and Toca FC treatment vs lomustine or temozolomide or bevacizumab	Glioblastoma Multiforme or Anaplastic Astrocytoma	Phase 2/3: Terminated (*n* = 403)	Tocagen Inc	Toca 511 and Toca FC treatment did not show better show better efficacy than the standard of care treatment used in the study. This trial was subsequently terminated by Tocagen Inc

^a^Status as on 12/6/2022 based on clinicaltrials.gov

Few examples of clinical trials based on replication-defective adenoviral vectors harboring suicide genes are discussed here. A phase I clinical trial studied the toxicity and efficacy of Ad5.SSTR/TK.RGD [NCT00964756] in patients with recurrent gynecologic cancers. It is an adenoviral vector carrying HSV-TK suicide gene and a somatostatin receptor (SSTR) construct for non-invasive imaging of gene transfer using In-111 pentetreotide. 12 patients were administered with 1 × 10^9^–1 × 10^12^ vp/dose followed by GCV. This study demonstrated that Ad5.SSTR/TK.RGD vector was safe with potential efficacy and imaging capability [124]. Similarly, the efficacy of adenoviral vector armed with HSV-TK suicide gene (AdHSV-TK) was evaluated in patients with recurrent ovarian cancer in a Phase 1 trial. AdHSV-TK was administered at doses ranging from 1 × 10^9^—1 × 10^11^ plaque-forming unit (PFU) followed by GCV. This study demonstrated the safety of AdHSV-TK with GCV in women with ovarian cancer [125]*.* The safety and feasibility of intratumoral injection of another adenoviral vector encoding HSV-TK (Ad.TK) in advanced hepatocellular carcinoma (HCC) was evaluated in a phase I trial [NCT00844623]. 10 patients were administered with 10^10^—2 × 10^12^ vp followed by GCV. This study demonstrated that intratumoral injection of Ad.TK was safe in HCC patients at the highest dose tested [126]*.*

While majority of adenoviral vector based clinical trials utilized replication defective vectors, several institutes including ours have developed replication-competent adenoviral vectors. Some of these studies are discussed in this section to give perspective to the trials conducted at Henry Ford Health. VCN-01 is an Ad5 based oncolytic adenovirus engineered to replicate in RB1 pathway dysfunctional cancer cells and expresses PH20 hyaluronidase. The safety of VCN-01 was evaluated in a phase I trial [NCT02045602] in advanced solid cancers in combination with gemcitabine or nab-paclitaxel. This study was found to be safe with indications of viral replication and an increase in levels of immune biomarkers was also observed [127]. Similarly, preclinical studies on replication-competent, tumor selective oncolytic adenovirus DNX-2401 (Delta-24-RGD or tasadenoturev) demonstrated favorable anti-tumor effects in glioma cells [128, 129]. This viral vector was successfully used in a Phase 1, dose escalation and biologic end point study in recurrent malignant glioma. The biologic end point group patients demonstrated that DNX-2401 replicated and spread within the tumor indicating direct oncolysis by the virus. Radiographical and histopathological examination demonstrated signs of inflammation and tumor infiltration by the immune cells [130]*.* The safety of ICOVIR-5 (Ad-DM-E2F-K-Delta24RGD) which derived from DNX-2401 was evaluated in a Phase I dose escalation trial [NCT01864759] in metastatic melanoma patients. This clinical study demonstrated the ability of ICOVIR-5 to reach melanoma metastases with a single intravenous administration [131]. ONCOS-102 (Ad5/3-D24-GMCSF) is an oncolytic adenovirus coding for granulocyte–macrophage colony-stimulating factor (GM-CSF) modified to replicate selectively in p16/Rb-defective cells. In ONCOS-102, GM-CSF is produced in a tumor-restricted manner since GMCSF is under viral E3 genes and the replication of the virus is tumor-selective. In a phase 1 study the safety, optimal dose, tolerability, and adverse event (AE) of ONCOS-102 was evaluated in patients with treatment refractory solid tumors. Favorable efficacy in terms of viral transduction and tumor cell killing was observed with no AE. This study established the safety and potential of ONCOS-102 in treating cancer patients [132]*.* All these preclinical and clinical studies demonstrate how far the gene therapy field has moved recently and clear advantages of deploying replication competent adenoviruses in cancer treatment paradigm.

## Challenges and future directions

The earliest clinical studies on gene therapy started with the first human gene therapy trial conducted in 1989 in patients with advanced melanoma [133]. Out of the approximate 2597 clinical trials on gene therapy conducted worldwide by 2017, more than 65% were designed for cancer management. These trials have paved the way for approvals of various gene therapy products for cancer management (Table 2) [134]. However, this number may seem small when compared with the large number of clinical trials that have been conducted. One of the main challenges impeding clinical translation of cancer gene therapy trials is that patients that are enrolled in trials already have advanced and therapy resistant cancers. Gene therapies may show better results in patients with early-stage cancers or when used as an adjuvant therapy after radiation or chemotherapy with potential for enhanced results. Including a wide range of patients as well as tumor genomic analysis, and evaluation of host immune response may help in improving selection of gene therapy most appropriate for the patients. Other challenges include the limited number of genes that can be used in clinical trials for cancer gene therapy, inefficient vectors, development of treatment resistance leading to recurrence of tumor and shorter survival of patients, lack of success in targeting metastatic cells etc. [35, 135]. There are several hurdles to overcome in the use of oncolytic viral vectors in cancer gene therapy such as issues with virus delivery, the immunosuppressive TME and anti-viral immune responses, tumor heterogeneity, etc. Improvements in virus delivery and distribution, tumor targeting, increasing potency of OV by incorporating therapeutic genes, and using OV in combination with other therapy approaches may help overcome some of the current challenges in OV [58]*.*There is also a need to analyze the current state of cancer gene therapy strategies with better technological advancements and more clarifying preclinical studies [5]. Developing new experimental vectors, increasing the specificity and efficiency of gene delivery system and better understanding of cancer and the immune response to therapy are areas in cancer gene therapy that can be improved [136, 137]. With time, these techniques can advance to be utilized as standalone treatment or in combination with current therapy approaches to manage cancer more efficaciously.

## Summary

One of the advantages of gene therapy over conventional therapy for cancer treatment is the inherent safety of the approach leading to tolerable side effects. Cancer gene therapy has novel modes of action, targeting specific mechanisms of cell death, and shows synergy with conventional therapeutic approaches. Improvement in gene therapy that can facilitate a better selection of appropriate patients for gene therapy include tumor genomic analysis, and assessment of host tumoral and cellular immunity. Developing safe and effective vectors for gene delivery and understanding the activity of nucleases can facilitate future genome editing as new treatment approaches for cancer.

Over the past two decades Henry Ford Health has developed replication competent oncolytic adenovirus gene therapy vectors and carried out several clinical trials with encouraging results in prostate and pancreatic cancers [86–88, 90, 108]. The Henry Ford Health team recently initiated a phase I trial in recurrent glioma. The first-generation adenoviral vector Ad5-CD/TK*rep* showed enhanced cancer cell killing with 5-fluorocytosine and ganciclovir prodrug therapy and sensitized cancer cells to radiation in vitro. The improved second-generation virus Ad5-yCD/*mut*TK_SR39_*rep*-ADP demonstrated good efficacy and safety profile alone [88, 108] or in combination with radiation [88, 90, 108, 109]. The third generation IL-12 incorporated vector Ad5-yCD/*mut*TK_SR39_*rep*-hIL-12 developed to target metastatic tumors showed good efficacy and safety in preclinical studies and was found to be tolerable in metastatic pancreatic cancer patients in a phase I clinical trial [89]. An additional gene therapy product, the Ad5-yCD/*mut*TK_SR39_r*ep*-hNIS adenoviral vector, incorporated an imaging gene, allowing for clinicians to monitor the activity of gene therapy vectors in vivo non-invasively. A vector with a reporter gene has the potential to improve the efficacy of the approach by monitoring activity from the injection site and, in addition, the safety of the approach by monitoring viral dissemination and persistence.

Apart from validating in-house developed gene therapy vectors [86–94], Henry Ford Health has been actively participating in numerous sponsored gene therapy clinical trials [138–143]. Collectively, these studies have demonstrated safety, tolerability and in some cases efficacy of gene therapy vectors. Considering the multiple clinical trials underway worldwide, the future of gene therapy remains bright with cautious optimism that gene therapy offers the potential to be part of an effective and safe treatment of cancer.

## Conclusions

Over the last three decades significant progress has been made in cancer gene therapy as evident by multitude of currently open clinical trials (Table 1) and approval of about a dozen gene therapy products for clinical interventions for cancer management (Table 2). Majority of FDA and EMA approvals for cancer management were granted within the last 10–12 years further highlighting the rapid pace at which the cancer gene therapy field is moving and providing the novel, cutting-edge treatment options to cancer patients who may have limited options given cancer progression on prior conventional therapies. Most of the presently FDA approved gene therapy products are for relapsed or refractory, or therapy resistant cancers. We are certain that the gene therapy will come out of age in the coming years where gene therapy products will be approved as first line therapy for cancer management. Exciting research and development taking place in gene therapy will make the next decade of biomedical research as a decade of “cell and gene therapy”.

## Data Availability

Not applicable.

## Abbreviations

OV: Oncolytic virotherapy
Ad: Adenovirus
TK: Thymidine kinase
CD: Cytosine deaminase
hNIS: Human sodium iodide symporter
GCV: Ganciclovir
5FC: 5-Fluorocytosine
HSV: Herpes simplex virus
EBRT: External beam radiotherapy
fSRS: Fractionated stereotactic radiosurgery
MTD: Maximum tolerated dose
DLT: Dose limiting toxicity
